# Micro-Expression Recognition Based on Pixel Residual Sum and Cropped Gaussian Pyramid

**DOI:** 10.3389/fnbot.2021.746985

**Published:** 2021-12-20

**Authors:** Yuan Zhao, Zhuang Chen, Song Luo

**Affiliations:** School of Computer Science and Engineering, Chongqing University of Technology, Chongqing, China

**Keywords:** micro-expression recognition, deep learning, Gaussian pyramid, pixel residual sum, position embedding

## Abstract

Facial micro-expression(ME) recognition has great significance for the progress of human society and could find a person's true feelings. Meanwhile, ME recognition faces a huge challenge, since it is difficult to detect and easy to be disturbed by the environment. In this article, we propose two novel preprocessing methods based on Pixel Residual Sum. These methods can preprocess video clips according to the unit pixel displacement of images, resist environmental interference, and be easy to extract subtle facial features. Furthermore, we propose a Cropped Gaussian Pyramid with Overlapping(CGPO) module, which divides images of different resolutions through Gaussian pyramids and crops different resolutions images into multiple overlapping subplots. Then, we use a convolutional neural networks of progressively increasing channels based on the depthwise convolution to extract preliminary features. Finally, we fuse preliminary features and make position embedding to get the last features. Our experiments show that the proposed methods and model have better performance than the well-known methods.

## 1. Introduction

Facial expression is a crucial channel for interpersonal socializing and can be used to convey inner emotions in daily life. Facial expression is divided into micro-expression(ME) and macro-expression. In past decades, macro-expression had a wide range of applications, and scholars have done a lot of research on macro-expression and facial recognition (Boucenna et al., 2014; Liu et al., 2018; Kim et al., 2019; Xie et al., 2019), but macro-expression is deceptive and can be easily hidden by human control. In contrast, ME will be unintentionally exposed as long as people intend to hide their true feeling. Hence, ME recognition has attracted much attention and has an extensive application prospect, such as clinical diagnosis, judiciary authorities, political elections, and national security.

ME has the following characteristics:

- ME is a very short facial expression and lasts between 1/25 and 1/3 (Yan et al., 2013). As a result, untrained individuals have a weaker ability to recognize ME (Lies, 1992).
- ME is an unconscious and involuntary facial expression appearing when people disguise one's emotions and can be triggered in high-risk environments and show real or hidden emotions.
- ME usually only appears in specific locations (Ekman and Friesen, 1971; Ekman, 2009b).
- ME usually needs to be analyzed in the video clip, and macro-expression can be analyzed in the image.

Due to these characteristics, it is difficult to recognize the ME artificially. Therefore, Ekman and Paul tried a lot of efforts to improve the ability of individuals to recognize the ME, and they developed a tool for ME recognition in 2002 Micro Expression Training Tool (METT) (Ekman, 2009a), which can effectively improve the individual's ability to recognize ME. However, the accuracy of relying on human recognition of ME is not high. According to reports, the accuracy of human-identified ME is only 47% (Frank et al., 2009). Therefore, it is particularly important to recognize the ME through computer vision. With the development of technology, the rise of high-speed cameras and deep learning has made it possible to accurately recognize the ME. However, the current ME recognition is mainly faced with the following problems.

- How to extract the subtle feature of the human face?
- How to overcome frame redundancy in the ME video?
- How to have stronger universality and overcome environmental changes?

The structure of the study is as follows: In Section II, the pieces of literature related to ME recognition are reviewed in detail; In Section III, a preprocessing method and network framework for ME recognition are proposed; In Section IV, we describe the experimental settings and analyze the experimental results; Finally, Section V summarizes this study with remarks. The contributions of this study are as follows.

- We propose two more effective methods of preprocessing, which combine spatio-temporal dimensionality and can extract more robust features.
- We design a module of Cropped Gaussian Pyramid with Overlapping(CGPO), which can use different scales information.
- We design a network with feature fusion, and the network structure adopts a gradual way of increasing channels.

## 2. Related Work

### 2.1. Handcrafted Features

Several years before, ME recognition was mainly based on traditionally handcrafted feature descriptors. These descriptors can be divided into geometric features and appearance features.

#### 2.1.1. Appearance-Based Features

For instance, Local Binary Pattern histograms from Three Orthogonal Planes (LBP-TOP) (Zhao and Pietikainen, 2007), Spatiotemporal Completed Local Quantization Patterns (STCLQP) (Huang et al., 2016), and LBP with Six Intersection Points (LBP-SIP) (Wang et al., 2014) can be considered as methods based on appearance features. These methods led that the features, dimensions are relatively high with more redundant information.

The LBP-TOP, a development of the LBP in a three-dimensional space, is a typical LBP descriptor with spatial-temporal characteristics. The LBP-TOP operator extracts LBP features on the three orthogonal planes. Next, obtained results are stitched as the final LBP-TOP feature, since the video can be regarded as a cube in the three dimensions of x, y, and t. The LBP-TOP not only considers the spatial information but also considers the information in the video sequence. After obtaining the LBP-TOP features, Zhao et al. use Support Vector Machine(SVM) for spotting and classification. Zhao et al. made good use of LBP-TOP features, and used many tricks of conventional expression analysis. As an early work, the work has achieved good results and has established the basis for the subsequent ME recognition.

The LBP-TOP has great limitations for only considering the local appearance and movement characteristics. So, Huang et al. (2016) proposed STCLQP for the ME recognition. First, three significant information, including magnitude, orientation, and sign components, are extracted by STCLQP. Second, for each component in temporal and appearance domains, Huang et al. (2016) made dense and characteristic codebooks by developing productive codebook selection and vector quantization. Finally, in terms of this codebook, Huang et al. (2016) extracted and fused spatio-temporal features, included orientation components, magnitude, and sign. Compared with LBP-TOP, the STCLQP method considers more information. Although the recognition accuracy is improved, it will inevitably lead to higher dimensions.

Furthermore, Wang et al. (2014) proposed LBP-SIP volumetric descriptor, which is based on three intersecting lines passing through a central point. The superabundance of LBP-TOP patterns is diminished by LBP-SIP. Furthermore, LBP-SIP provides a more dense and weightless characterization and reduces computational complexity. It further promotes the improvement of the accuracy of the ME recognition and has become the baseline for many subsequent works.

#### 2.1.2. Geometric-Based Features

Optical flow, a geometric-based feature, calculates the displacement of facial feature points or the optical flow of the action area. It can extract representative motion features that are robust for the diversity of facial textures. Furthermore, the data except for RGB channels can be enhanced by optical flow (Liu et al., 2019).

Many works treat optical flow as a data preprocessing step. Liu et al. (2015) proposed an uncomplicated yet productive Main Directional Mean Optical-flow (MDMO) feature. On the ME video clips, an effective optical flow method is adopted. Meanwhile, Liu utilizes partial action units to divide the face into regions of interest (ROIs). MDMO is a normalized feature based on ROIs. It combines both spatial location and local statistic motion characteristics. MDMO has the advantage of small feature dimensions.

Some works (Liong et al., 2019; Liu et al., 2019; Zhou et al., 2019) utilized optical flow information for ME recognition and have achieved good results. For instance, Liu et al. (2019) utilized two domain adaptation methods, which include expression magnification and reduction and adversarial training. Then, he preprocessed the raw images to capture the spatio-temporal optical flow from facial movements from onset frame (the first frame in the ME video) to apex frame (the most intense frame of action in the ME video), won the championship of 2019-the second facial Micro-expressions Grand Challenge (MEGC2019) (See et al., 2019). Zhou et al. (2019) captured the TV-L1 optical flow (Zach et al., 2007) of the onset frame and the mid-position frame, and then performs ME recognition through the Dual-Inception network. Instead of using apex frames, they use mid-position frames to cut down computation complexity. Furthermore, Liong et al. (2019) designed a STSTNet, which can be used to learn three features of optical flow, namely vertical optical flow, optical strain, and horizontal optical flow. These features are calculated by the onset frame and apex frame of ME video.

Optical flow has the advantage of small feature dimensions and the ability to capture subtle muscle movements. However, the optical flow has higher requirements on light and is easily affected by the external environment. In addition, these works only use the optical flow information of the apex frame and onset frame and lose the motion information of other frames.

### 2.2. Deep Neural Networks

Deep learning (LeCun et al., 2015) is universally used in various industries. Especially during the immediate past, the works on deep learning in the ME recognition field has gradually increased. In the field of deep learning, the features preprocessed by the optical flow method and LBP can be used as the input of convolution neural network (CNN). Then, CNN is usually used for feature extractors. For instance, Xia et al. (2019) proposed spatio-temporal recurrent convolutional networks based on optical flow, which extracts the optical flow information from the onset frame until the apex frame and inputs it into recurrent convolutional networks.

Furthermore, some works also use Long Short-term Memory (LSTM) to directly input ME video clips. One early work (Khor et al., 2018) proposed an Enriched Long-term Recurrent Convolutional Network (ELRCN). First, every ME frame is encoded into a feature vector by CNN modules. Then, ELRCN uses an LSTM module to pass the feature vector and predicts ME at last. ELRCN uses the feature that the information can be retained for a long time in the gating unit to detect ME in the video, and achieve good performance. Therefore, the combination of LSTM and CNN have greater advantages in recognizing ME in videos. However, due to the small changes in the ME video clips, there is frame redundancy, leading to greater computational complexity.

In conclusion, compared with traditional manual features for ME recognition, deep learning technology can extract features from ME videos and classify them with higher accuracy. However, due to frame redundancy in ME videos, the speed of the deep learning training model is greatly affected. Therefore, we propose two new ME video preprocessing methods to overcome frame redundancy in ME video and improve the recognition of ME classes.

## 3. Method

### 3.1. Preprocessing

**Figure 1:**
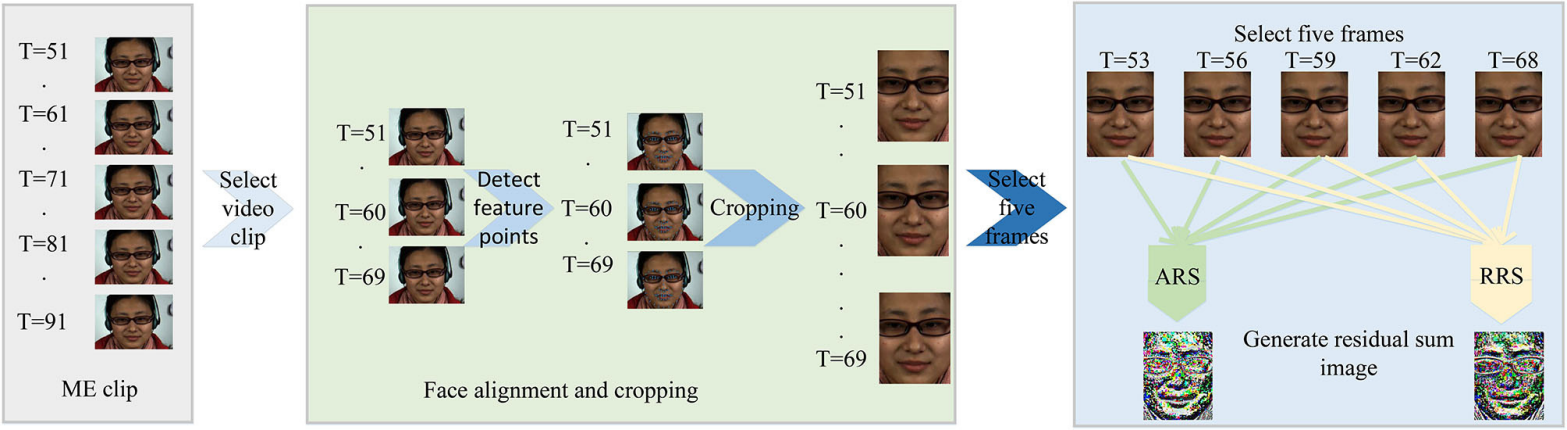
Preprocessing Flow chart (©Xiaolan Fu).

As we discussed above, it is an inevitable stage to extract a discriminative and efficient feature. Therefore, this study proposes two methods based on the residual sum of image pixels to extract salient features: (1) Absolute Residual Sum (ARS) and (2) Relative Residual Sum (RRS). These methods take the frames in the ME clip at fixed intervals and consider the regional pixel displacement between frames. It not only avoids the redundancy of the ME clip but also makes full use of the ME information. The pixel-level displacement difference sum, named RS, can explain the tiny movement of the object. ARS and RRS preprocessing procedure are shown in Figure 1.

#### 3.1.1. Absolute Residual Sum

Preprocessing is divided into five stages.

##### 3.1.1.1. Select Video Clip

He et al. proposed MDMD, which used a reciprocal change from the onset frame to the offset frame to spotting ME (He et al., 2020). Therefore, we only recognize the ME from the onset frame to the apex frame. First, we select a video clip from the ME video and calculate its start and end. We select the partial video clips from the ME video clip. The onset frame is taken as the start by Equation (1), and select the end by Equation (2).


(1) $$\begin{array}{l} start=T\left(onset\right) \end{array}$$

Where T(*x*) represents the frame sequence of x in the video.


(2) $$\begin{array}{l} end=\{\begin{array}{ll} min\left(T\left(onset\right)+10,T\left(offset\right)\right) & if\;T\left(apex\right)-T\left(onset\right)\\ \; & <10\\ min\left(T\left(apex\right),T\left(offset\right)\right) & else \end{array} \end{array}$$

Where *min*(*x, y*) represents the smaller values of *x* and *y*.

##### 3.1.1.2. Detect Feature Point

The dlib library is utilized to spotting facial feature points.

##### 3.1.1.3. Cropping

Cropping the face through the face feature points.

##### 3.1.1.4. Select Five Frames

Notice that, ME data is very redundant. Useful information must be mined from the data. A few other works (Li et al., 2013; Le Ngo et al., 2015, 2016) have proposed many methods to reduce frame redundancy in ME videos by using partial frames. Therefore, we require mining crucial frames from ME video clip. We define crucial frames as key-frames and define frames except for the key-frames as transition frames. Furthermore, we make two assumptions for getting rid of transition frames: (1) Transition frames are highly similar to the key-frames, and deletion does not affect the recognition accuracy. (2) Transition frames are continuously distributed, centered on key-frames.

Hence, we choose appropriate intervals by Equation (3) and select five key-frames as elements in 𝔽 according to Equation (4).


(3) $$\begin{array}{l} gap=\left\lceil \frac{end-start}{N_{key}+1}\right\rceil \end{array}$$

(4) $$\begin{array}{l} 𝔽=\left\{min\left(start+gap,end\right),min\left(start+gap*2,end\right),...,end\right\} \end{array}$$

Where ⌈*x*⌉ is taking the smallest integer not less than *x* for some scalar, and *N*_*key*_ represents the number of key frames. *N*_*key*_ is set to five in the paper.

##### 3.1.1.5. Generate Redisual Sum Image

Liu et al. (2019) took the motion difference between the onset frame and each frame to calibrate the apex frame, because the intensity relationship of ME can be indicated by the motion difference. Therefore, we cumulate the motion difference for calculating the variation trend of a single pixel. For the key frame in 𝔽, Equation (5) is used to calculate the ARS.


(5) $$\begin{array}{l} ares\left(x,y,z\right)=\left(\underset{f\in 𝔽}{\sum }\left(\left|Q_{f}\left(x,y,z\right)-Q_{start}\left(x,y,z\right)\right|\right)\right)\;\%\;256 \end{array}$$

Where *Q*_*f*_(*x, y, z*) represents the pixel value of the three-channel image (*x, y, z*) of the *f*_*th*_ frame and *ares*(*x, y, z*) represents the pixel value of the generated ARS image.

#### 3.1.2. Relative Residual Sum

As shown in Figure 1, the steps before the fifth step are the same as ARS. In the fifth step, we use Equation (6) to calculate the sum of residuals between frames. Then, we use Equation (7) to transform the range of sum to between *gmin* and *gmax*. In this experiment, *gmin* = 0 and *gmax* = 255.


(6) $$\begin{array}{l} diff\left(x,y,z\right)=\left(\underset{f\in 𝔽}{\sum }\left(\left|Q_{f}\left(x,y,z\right)-Q_{start}\left(x,y,z\right)\right|\right)\right) \end{array}$$

(7) $$\begin{array}{l} rres\left(x,y,z\right)=\frac{\left(diff\left(x,y,z\right)-min\left(diff\right)\right)}{max\left(diff\right)-min\left(diff\right)}*\left(gmax-gmin\right)+gmin \end{array}$$

Where *max*(*x, y*), *diff*(*x, y, z*), and *rres*(*x, y, z*) represent the greater values of *x* and *y*, the sum of the displacement of the video frame at the three-channel image (*x, y, z*), and the pixel value of the generated RRS image, respectively.

### 3.2. Framework

CropNet, based on the depthwise convolution (Sandler et al., 2018), is used as a classification model. CropNet takes advantage of CGPO. The architecture of the CropNet is shown in **Figure 3**. Conv, BN, and FC in the figure represent Convolutional Layer, Batch Normalization Layer, and Fully Connected Layer, respectively.

#### 3.2.1. Image Augmentation

**Figure 2:**
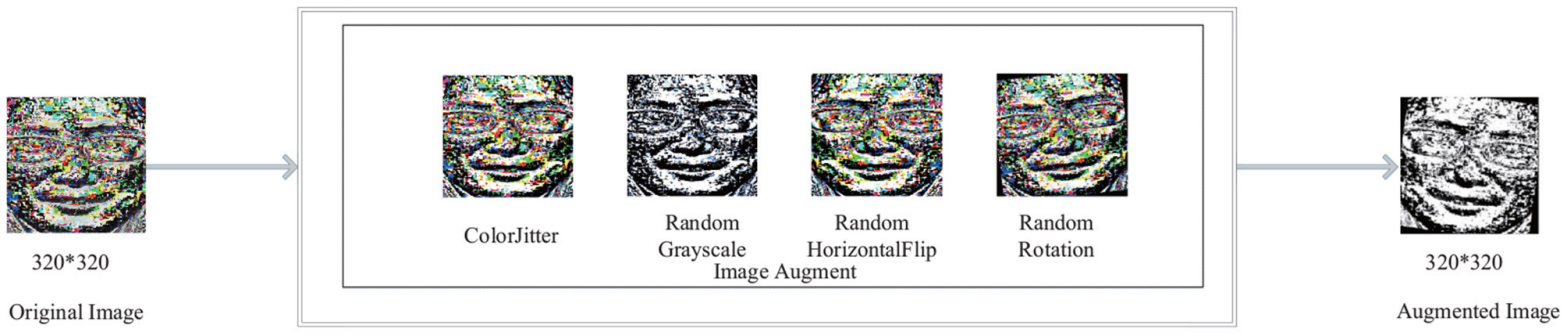
Image augment module.

The number of network parameters is approximately 7.6M. Image augment is essential as the network framework is slightly large. According to the characteristics of the human face, we performed the following four data augmentation in turn. (1) The image brightness, contrast, and saturation are randomly changed to [20%, 180%] of the original image brightness, and the hue offset of the image is changed to [−0.5, 0.5] of the original image. (2) The picture is converted to grayscale with a probability of 20%. (3) Flipping the image horizontally with a 50% probability. (4) Rotating the image randomly clockwise [−15,15] degrees. The image augment module is shown in Figure 2.

#### 3.2.2. Cropped Gaussian Pyramid With Overlapping

**Figure 3:**
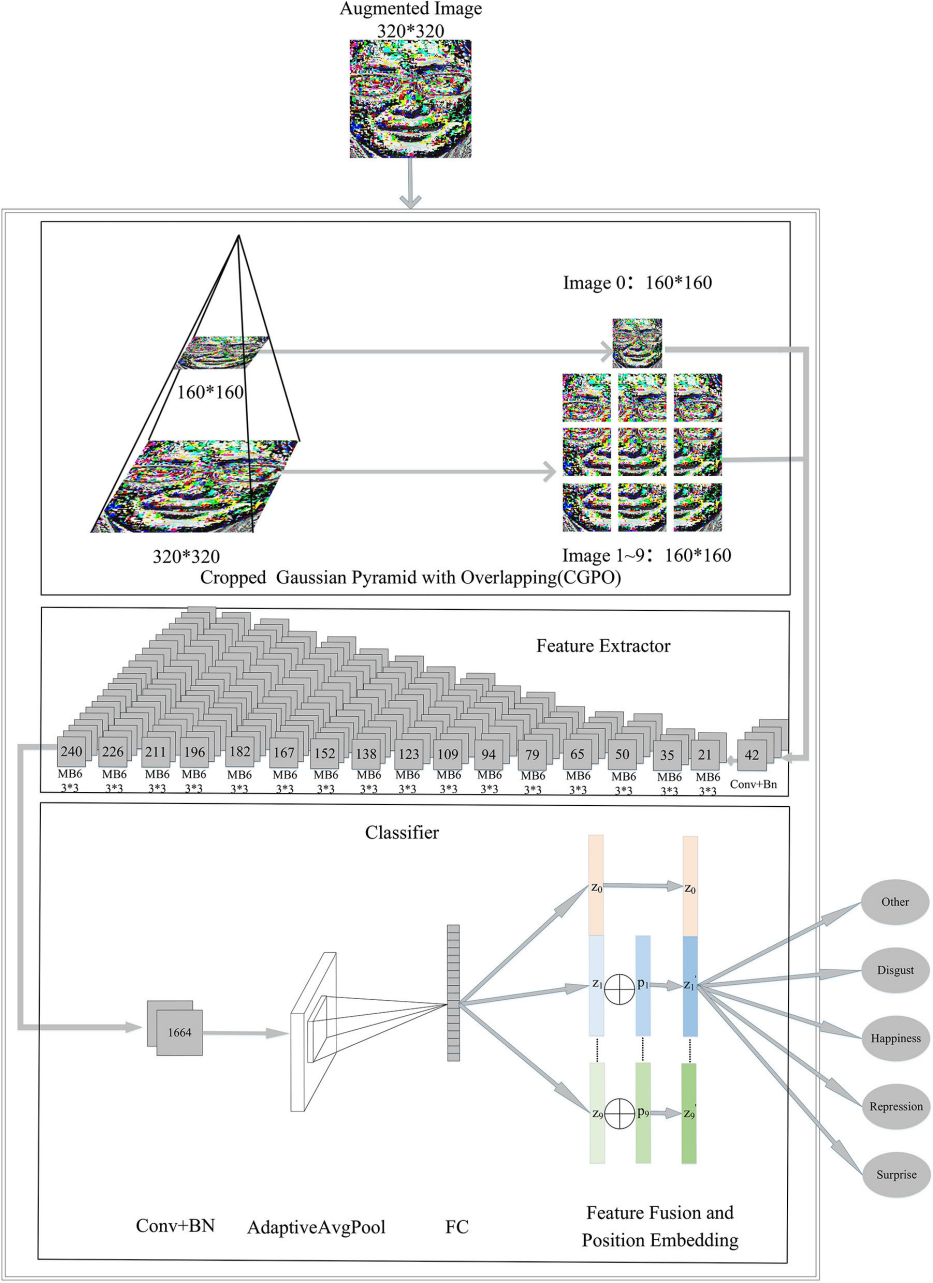
The architecture of the network model. The numbers on convolution and MB6 block represent the number of output channels. MB6 refers to MobileNetV2 (Sandler et al., 2018)'s inverted bottlenecks with an expansion ratio of 6.

Different facial areas have different importance in the production of ME. Therefore, we propose a CGPO module, which divides ME video frames with different resolutions of the image into 10 overlapping subplots. It can separate the mouth, the eyes, the nose, etc. The introduction of overlapping mechanisms can reduce the risk of separating important parts of the face. The CGPO module is shown in Figure 3 CGPO, and its processing flow is as follows.

- First, we require 320 × 320 resolution of the image input and down-sample it to get an image with a resolution of 160 × 160.
- Second, for each image with different scale resolution, we divide them into several 160 × 160 images and introduce the overlap factor α. α is used to control the size of the overlap when crop images with different precision. In this study, α is 0.3.
- Finally, after going through the above process, images are fed CNN based with the depthwise convolution.

#### 3.2.3. Feature Extraction

Han et al. (2020) designed ReXNet, which has achieved very good results in the ImageNet Challenge. Therefore, we use the ReXNet feature extraction module as the extractor. A network of progressively increasing channels are leveraged on the extracting feature, as shown in Figure 3 feature extractor.

Due to the difficulties in data collection and identification of ME, there are few ME datasets. It is difficult to apply deep learning in ME recognition. Therefore, we train this module on the ImageNet datasets (Deng et al., 2009) and then apply it to the ME recognition through the transfer learning method (Pan and Yang, 2009).

#### 3.2.4. Feature Fusion and Classifier

Feature Fusion and Classifier are shown in Figure 3 Classifier. The features extracted in the previous module go through the Convolutional Layer, Batch Normalization Layer, Adaptive Pooling Layer, and Fully Connected Layer, in turn, and become a feature vector $z_{i}\in {\mathbb{R}}^{24}$, where i represents the order of segmented images. Since the CGOP module segmented a total of 10 images, we could obtain 10 feature vectors {***z***_**0**_, ***z***_**1**_⋯⋯⋯***z***_**9**_}.

However, because the position information after image cropping becomes blurred, the model has a hard time learning about correlations between images. We combine the location information with the feature to make the features more explanatory. Therefore, for feature vectors {***z***_**1**_, ***z***_**2**_⋯⋯⋯***z***_**9**_} of segmented images, we introduce trainable position embedding vectors {***p***_**1**_, ***p***_**2**_⋯⋯⋯***p***_**9**_} to learn the position information of the image, where *p*_*i*_ has the same dimension as ***z***_***i***_. The position embedding vectors are initialized to random values that follow a normal distribution. The mean of the random values is 0 and the variance is 0.2. As shown in Equation (8), we calculate the new feature vectors $\left\{{z_{1}}^{\prime },{z_{2}}^{\prime }\cdots \cdots \cdots {z_{9}}^{\prime }\right\}$.


(8) $$\begin{array}{l} {z_{i}}^{\prime }=z_{i}⊕p_{i}\;0<i<10 \end{array}$$

Finally, we mix $\left\{z_{0},{z_{1}}^{\prime },{z_{2}}^{\prime }\cdots \cdots \cdots {z_{9}}^{\prime }\right\}$ by splicing and classifying ME.

## 4. Experiment

### 4.1. Datasets

Due to the characteristics of ME and its difficulty in triggering and collecting, the dataset is very scarce. As far as we know, there are three spontaneous datasets generally utilized for ME recognition: SMIC-HS (Li et al., 2013), SAMM (Davison et al., 2016), and CASME II (Yan et al., 2014a). The details of these three spontaneous datasets are shown in Table 1.

**Table 1:** Micro-expression (ME) datasets.

**Datasets**	**CASME II**	**SMIC-HS**	**SAMM**
Particpants	26	16	29
Samples	255	157	159
Resolution	640*480	640*480	960*650
Frame rate(fps)	200	100	200
FACS coded	✓	x	✓
APEX index	✓	x	✓
Emotion	Other(99) Disgust(63) Surprise(28) Repression(27) Sadness(4) Happiness(32) Fear(2)	Negative(66) Positive(51) Surprise(40)	Other(26) Happiness(26) Disgust(9) Surprise(15) Sadness(6) Anger(57) Fear(8) Contempt(12)

### 4.2. Experiment Settings

All experiments for this study were all carried out on Ubuntu 16.04 and Python 3.6.2 with Pytorch 1.6 on NVIDIA GTX Titan RTX GPU (24 GB). The label smoothing loss function (Lukasik et al., 2020) is leveraged as the loss function. It can better generalize the network and ultimately produce, more accurate predictions on invisible data. AdamP (Heo et al., 2021) is used as an optimizer. We use UF1 (commonly referred to as the macro average F1 score), UAR (commonly referred to as balanced accuracy), and Accuracy as our evaluation standard.

- **UF1** score can equally emphasize in a rare class. So, it is a suitable indicator in a multi-class evaluation. The calculation formula for UF1 is as follows:
  (9) $$\begin{array}{l} UF1=\frac{1}{C}\overset{C}{\underset{i=1}{\sum }}\left(\frac{2*TP_{i}}{2*TP_{i}+FP_{i}+FN_{i}}\right) \end{array}$$
  Where *C* represents the number of classes and *FP*_*i*_, *TP*_*i*_, and *FN*_*i*_ represent the false positive, the true positive, and the false negative for the *i*_*th*_ class, respectively.
- **UAR** is a more appropriate indicator instead of the standard accuracy indicator that may be partial to larger classes. The calculation formula for UAR is as follows:
  (10) $$\begin{array}{l} UAR=\frac{1}{C}\overset{C}{\underset{i=1}{\sum }}\left(\frac{TP_{i}}{N_{i}}\right) \end{array}$$
  Where *N*_*i*_ represents the number of *i*_*th*_ class.
- **Accuracy** is commonly used as a CASME II experiment in five classes. The calculation formula for Accuracy is as follows:
  (11) $$\begin{array}{l} Accuracy=\frac{TP}{N} \end{array}$$

### 4.3. Experiment With Five Classes of ME in the CASME II

We choose the CASME II as the evaluation dataset. Only five classes (Others, Disgust, Happiness, Repression, and Surprise) are considered, since the fear and sadness samples are very scarce. In this experiment, Leave-One-Subject-Out (LOSO) cross validation is utilized for evaluation protocol. LOSO cross validation refers to using the samples of one subject as the test set, and the rest as the training set in each fold. It can prevent the test set and the training set from having the same sample, thereby avoiding data leakage. Recognition Accuracy can be calculated by the LOSO cross validation evaluation protocol. In the same evaluation standard, we compare with a variety of methods. The result is shown in Table 2.

**Table 2:** Comparison of ME recognition performance in CASME II (5 classes).

**Method**	**Accuracy**
LBP-Top+AdaBoost (Le Ngo et al., 2014)	0.437
STCLQP (Huang and Zhao, 2017)	0.583
ELRCN (Khor et al., 2018)	0.524
DSSN (Khor et al., 2019)	0.707
TSCNN-I (Song et al., 2019)	0.740
SSSN (Khor et al., 2019)	0.711
TSCNN-II (Song et al., 2019)	0.810
Bi-WOOF (apex and onset) (Liong et al., 2018)	0.578
Su et al. (Su et al., 2021)	0.727
**RRS+CropNet(ours)**	0.790
**ARS+CropNet(ours)**	**0.862**

**Figure 4:**
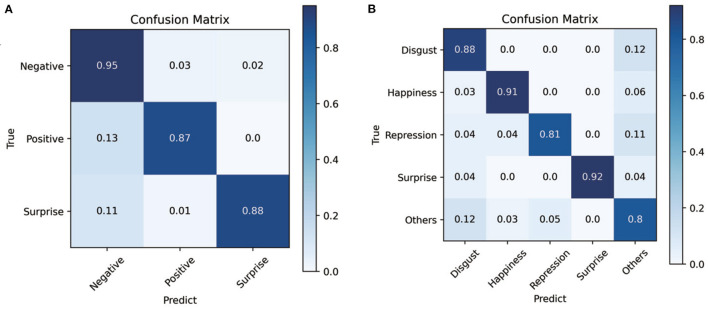
**(A)** is the confusion matrix of composite datasets (SMIC-HS, CASME II, and SAMM) in the absolute residual sum (ARS) and the CropNet. **(B)** is the confusion matrix of CASME II in the ARS and the CropNet.

The confusion matrix obtained by applying the ARS and the CropNet is shown in Figure 4B. Through the confusion matrix, the overall recognition rate is very high. The proposed method has great performance for all classes.

### 4.4. Composite Datasets Evaluation (CDE)

Composite datasets evaluation is a very effective evaluation method in cross-database ME recognition. In this experiment, we use the MEGC2019 standard. According to MEGC2019 standards, we combined all samples of the datasets (SAMM, CASME II, and SMIC-HS) into a composite dataset by unifying the number of ME class. ME are divided into three classes: negative, surprised, and positive. Disgust, contempt, fear, sadness, and anger is regarded as the negative class. Surprise is still regarded as surprise class. Happiness is regarded as the positive class. LOSO cross validation is utilized to split the training set and test set. Table 3 compares the performance of proposed methods against a number of recent study. The methods in Table 3 were all compared in the same datasets and at the same evaluation standard. The confusion matrix obtained by applying the ARS and the CropNet is shown in Figure 4A. It shows that three classes have similar performance, and the proposed method also has a good fit for unbalanced data.

**Table 3:** Comparison of ME recognition performance composite datasets.

**Method**	**Composite**		**SMIC-HS**		**CASME II**		**SAMM**	
	**UF1**	**UAR**	**UF1**	**UAR**	**UF1**	**UAR**	**UF1**	**UAR**
LBP-TOP (Zhao and Pietikainen, 2007)	0.588	0.578	0.200	0.528	0.702	0.742	0.395	0.410
Bi-WOOF (Liong et al., 2018)	0.629	0.622	0.572	0.582	0.780	0.802	0.521	0.512
CapsuleNet (Van Quang et al., 2019)	0.652	0.650	0.582	0.587	0.706	0.701	0.620	0.598
OFF-ApexNet (Gan et al., 2019)	0.719	0.709	0.681	0.669	0.876	0.868	0.540	0.539
Dual-Inception (Zhou et al., 2019)	0.732	0.727	0.664	0.672	0.862	0.856	0.586	0.566
STSTNet (Liong et al., 2019)	0.735	0.760	0.680	0.701	0.838	0.868	0.658	0.681
ELTRCN (Khor et al., 2018)	0.788	0.782	0.746	0.753	0.829	0.820	0.775	0.715
RCN-S (Xia et al., 2020)	0.746	0.710	0.651	0.657	0.836	0.791	0.764	0.656
STSTNet+GA (Liu et al., 2021)	0.836	0.836	0.814	0.812	0.882	0.891	0.800	0.790
**RRS+CropNet(ours)**	0.875	0.877	0.813	0.819	0.972	0.969	0.842	0.827
**ARS+CropNet(ours)**	**0.911**	**0.904**	**0.855**	**0.851**	**0.974**	**0.979**	**0.912**	**0.893**

Note that, the apex frame spotting is indispensable for ME recognition since the apex frame of the SMIC-HS dataset is not labeled. In recent years, there are a lot of apex frames spotting works (Yan et al., 2014b; Li et al., 2018; Peng et al., 2019; Zhou et al., 2019). In fact, apex frame spotting is a very difficult work. Therefore, this experiment considers a trade-off between efficiency and effectiveness. The middle frame of the video in the SMIC-HS dataset is used as the apex frame.

### 4.5. Ablation Experiments

We performed two ablation experiments on the CASME II dataset to verify the effectiveness of the module.

- We performed ablation experiments on preprocessing methods for comparing the effectiveness of the four preprocessing methods ARS, RRS, Farneback optical flow (Farnebäck, 2003), and TV-L1 optical flow.
- We performed ablation experiments on model architect for verifying the effect of the GCOP module.

As shown in Table 4, ARS stands out among the four preprocessing methods. It can extract more reliable spatio-temporal features and improve the UF1 value of ME recognition. RRS also achieves very good results. There are significant differences between these two methods. RRS pays more attention to areas with greater displacement by relative displacement change between unit pixels, while is not too sensitive to small displacement areas. ARS considers the trade-off between displacement regions of different scales, which can focus on both small displacement areas and large displacement areas. Therefore, subtle displacement can be captured. At the same time, for areas with frequent displacement, ARS ignores the displacement of unit pixels and pays attention to regional displacement. But in our experimental environment, Farneback optical flow and TV-L1 optical flow are far less effective than the proposed methods in this study.

**Table 4:** Ablation experiments in CASME II (5 classes).

**Ablation module**	**Ablation method**	**UF1**	**Accuracy**
paper method	**CropNet+ARS**	**0.863**	**0.862**
Preprocessing Method	CropNet+RRS	0.803	0.790
	CropNet+Optical FLow(Farneback)	0.661	0.625
	CropNet+Optical FLow(TV-L1)	0.697	0.669
Model architect	CropNet without GCOP +ARS	0.841	0.813

The Cropped Gaussian Pyramid with Overlapping module focuses on different areas of the face, extracts features for each area, and then stitches the obtained features to classify them. Through the ablation experiment in Table 4, it is easy to find the efficiency of the CGPO module and the ARS method.

**Figure 5:**
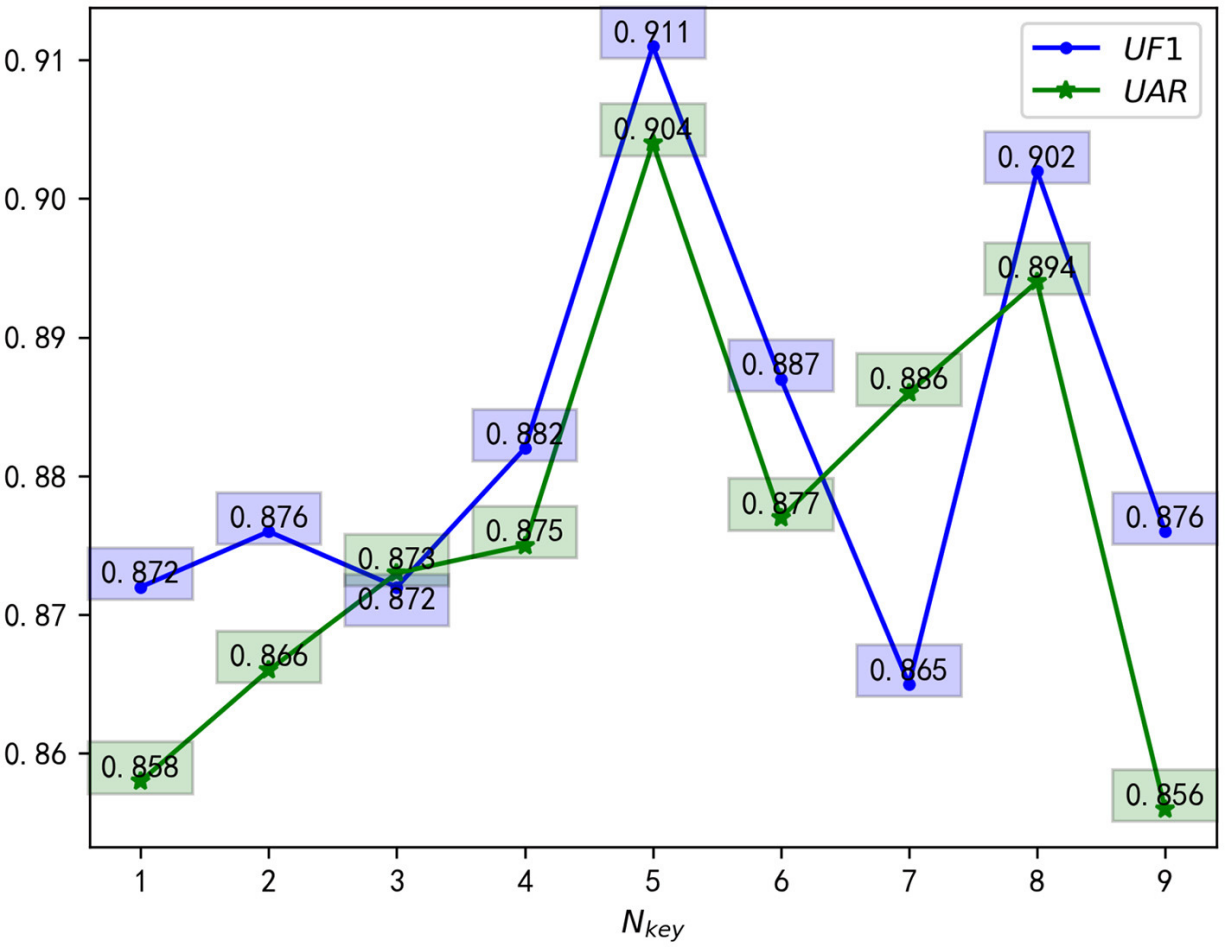
*N*_*key*_ hyperparameter's ablation experiments.

Furthermore, we conducted hyperparameter's ablation experiments in MEGC2019 composite datasets for verifying the effectiveness of the hyperparameters *N*_*key*_. The experimental results are shown in Figure 5, which can be concluded that there is greater universality when *N*_*key*_ is set to five. Therefore, in all experiments, we only select five key-frames at equal intervals in the ME video clip.

### 4.6. Visualization Experiments

**Figure 6:**
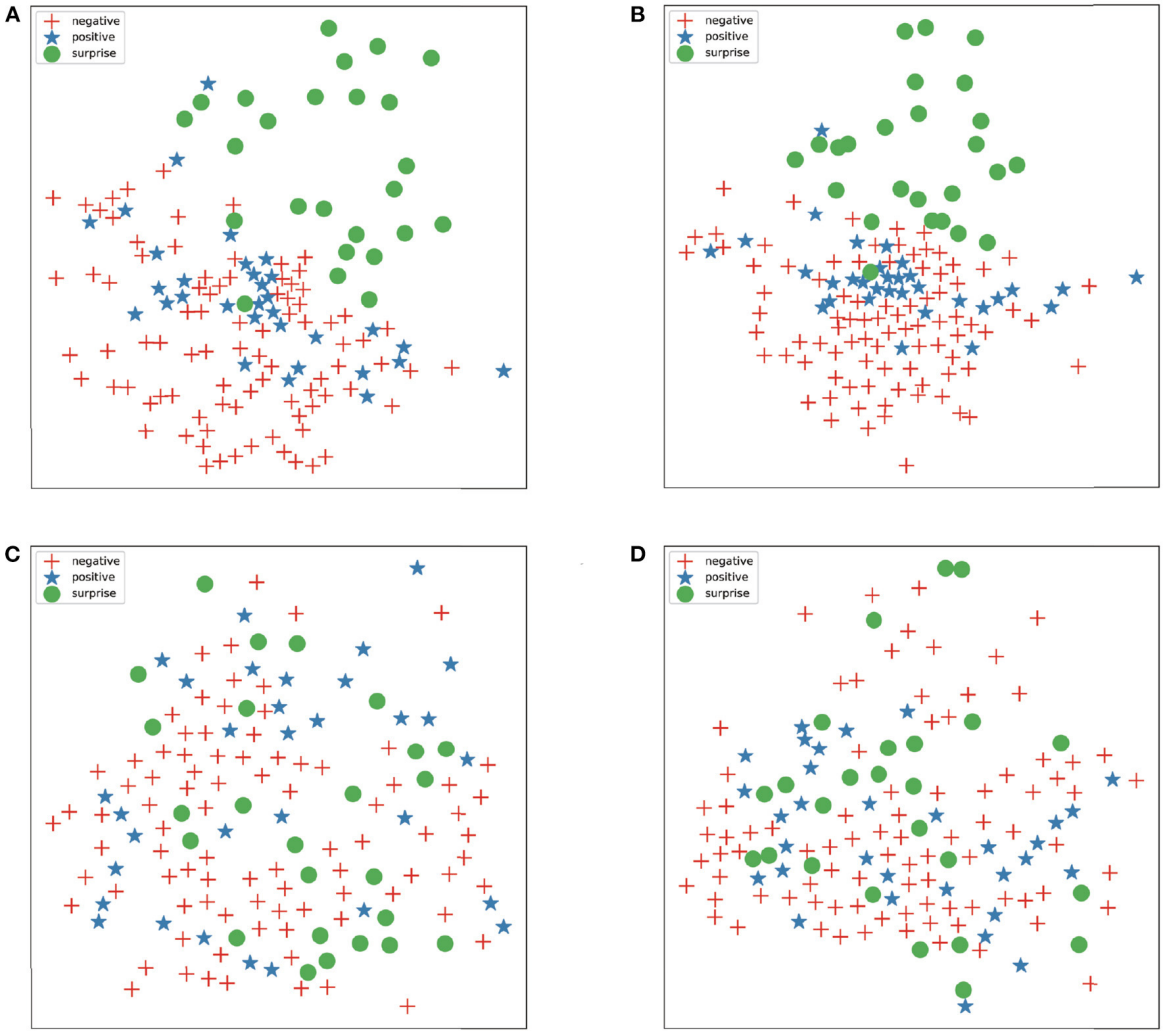
**(A–D)** represent preprocessing images by ARS, relative residual sum (RRS), farneback optical flow and TV-L1 optical flow, respectively.

We use T-SNE (Van der Maaten and Hinton, 2008) to visualize the preprocessed image for better comparing the effects of the proposed preprocessing methods. Figure 6 shows the feature distribution of images preprocessed by various methods. In this experiment, we use three classes (negative, positive, and surprised) of CASME II.

The features extracted using Farneback optical flow and TV-L1 optical flow are disorganized, but the image extracted by residual sum methods can already distinguish many features. For example, surprise ME is easy to distinguish from other expressions. After preprocessing by the residual sum method, the features become initially orderly, but some of the ME are still mixed together. Therefore, further extraction of features through CNN can enhance the validity of features.

## 5. Conclusion

In this study, we propose two novel preprocessing methods to solve ME recognition tasks with spatial-temporal feature extraction. These methods use the displacement residual sum of the unit pixels of the ME clip to extract a subtle motion feature. Through our experiment, it responds well to environmental change and subtle displacement. In addition, we propose a CGPO module, which divides the image into partial overlapping pictures of different precision and extracts features from different pictures. Hence, the model can focus on each facial local area, and then recognize the subtle movements of specific locations. Furthermore, we design CropNet which have a gradual way of increasing channels, features fusion module, and position embedding function.

In the experiment, we test the proposed two preprocessing methods and the designed network on the mixed dataset of MEGC2019 and five classes of ME on CASME II. The traditional manual method based on optical flow is labor-expensive and time-consuming, while the RRS and ARS preprocessing methods greatly improve the situation of frame redundancy and improve the recognition accuracy of each ME. In addition, the CGPO module can separate key parts of a person's face for more subtle feature extraction. In general, the method proposed in the study has better performance than the well-known method.

However, the proposed model does not belong to an end-to-end model, because it must go through the preprocessing method, which takes a certain amount of time to detect key points, align faces, crop, and calculate RRS and ARS. Therefore, in the future improvement, we will improve the method and model in this study into an end-to-end model.

## Data Availability Statement

The data analyzed in this study is subject to the following licenses/restrictions: this paper involves three databases (CASMEII, SMIC and SAMM). As each database involves human facial expressions, you need to apply for access. Requests to access these datasets should be directed to SMIC: Xiaobai.Li@oulu.fi, SAMM: M.Yap@mmu.ac.uk, CASMEII: eagan-ywj@foxmail.com.

## Author Contributions

YZ led the method design and experiment implementation. YZ and SL wrote sections of the manuscript. SL and ZC provided theoretical guidance, result review, and paper revision. All authors read and approved the final manuscript.

## Funding

This publication of this paper was supported by the National Natural Science Foundation of China (no. 61872051), the Scientific and Technological Research Program of Chongqing Municipal Education Commission of China (no. KJ1600932), and the Graduate Innovation Fund of Chongqing University of Technology (no. clgycx20203123).

## Conflict of Interest

The authors declare that the research was conducted in the absence of any commercial or financial relationships that could be construed as a potential conflict of interest.

## Publisher's Note

All claims expressed in this article are solely those of the authors and do not necessarily represent those of their affiliated organizations, or those of the publisher, the editors and the reviewers. Any product that may be evaluated in this article, or claim that may be made by its manufacturer, is not guaranteed or endorsed by the publisher.
